# Maternal physiological responses to oral neem leaf extract supplementation during late gestation in ewes

**DOI:** 10.1007/s11250-026-04939-5

**Published:** 2026-02-24

**Authors:** Mona M. M. Y. Elghandour, Armando Manglorio Parra García, Angela Gabriella D’Alessandro, Ekaette N. Mbaba, Emmanuel F. Istifanus, José Luis Ponce-Covarrubias, Abdelfattah Z. M. Salem

**Affiliations:** 1 Facultad de Medicina Veterinaria y Zootecnia, Universidad Autónoma del Estado de México, Toluca, Estado de México México; 2 Dirección General de Educación Tecnológica Industrial (DGETI), Cd. de, México; 3 Dipartimento di Scienze del Suolo, della Pianta e degli Alimenti (Di.S.S.P.A.), Università degli Studi di Bari, Via Giovanni Amendola, 165/a, Bari, 70126 BA Italy; 4 Department of Animal Science, University of Uyo, Uyo, Nigeria; 5 Escuela Superior de Medicina Veterinaria y Zootecnia, Universidad Autónoma de Guerrero, Técpan de Galeana, No. 3, Guerrero, México

**Keywords:** *Azadirachta indica*, Neem leaf extract, Pregnant ewes, Hematology, Biochemistry, Polyphenols, Late gestation

## Abstract

This study investigated the hematological and serum biochemical responses of pregnant ewes administered *Azadirachta indica* leaf extract (NE) during late gestation. Twenty-four pregnant ewes were assigned in a Completely Randomized Design to a 2 × 3 factorial arrangement comprising two sampling days (Day 1 and Day 30) and three Neem extract doses: 0 mL (NE0), 20 mL (NE20), and 40 mL (NE40), with three replicates per treatment. Blood samples were collected through the jugular vein on Days 1 and 30 and analyzed using an automated hematology analyzer and spectrophotometric kits. Data were subjected to analysis of variance using SAS 9.4, and means were separated by the Duncan multiple range test. Significant treatment effects were detected from the results for erythrocytes (*P* = 0.0187), mean corpuscular volume (*P* = 0.0032), glucose (*P* = 0.0002), total blood protein (*P* = 0.0269), monocytes (*P* = 0.0261), and eosinophils (*P* = 0.0453). Treatment × day interactions occurred for monocytes (*P* = 0.0261) and GGT (*P* = 0.0101). By Day 30, NE20 and NE40 ewes exhibited higher erythrocyte counts, reduced mean corpuscular volume, improved glucose preservation, and increased total protein relative to NE0, while liver and kidney biomarkers remained within physiological limits. These findings demonstrate that weekly administration of *A. indica* leaf extract at 20 mL/ewe is a safe and effective phytogenic strategy that enhances erythropoiesis, mitigates gestational hypoglycemia, improves protein status, and supports immune function without eliciting toxicity during late pregnancy.

## Introduction

Late gestation in ewes is characterized by profound physiological adaptations to meet high fetal nutrient demands, including expanded plasma volume leading to hemodilution, reduced erythrocyte indices, and progressive hypoglycemia driven by fetal glucose uptake and diminished maternal insulin sensitivity (Chikhaoui et al. 2023; Schlumbohm and Harmeyer 2008; Yaqub et al. 2021). These changes heighten susceptibility to oxidative stress, low-grade inflammation, and metabolic disorders such as pregnancy toxemia, particularly in multiple-bearing ewes (Schlumbohm and Harmeyer 2008; Yenilmez et al. 2021). Concomitant alterations in leukocyte profiles and liver enzyme activities reflect immune and hepatic adjustments in response to increased metabolic load (Yaqub et al. 2021; Chikhaoui et al. 2023). Phytogenic additives rich in polyphenols have emerged as promising interventions to counteract these challenges by scavenging reactive oxygen species, modulating inflammatory pathways, and enhancing glucose homeostasis and protein metabolism (Bešlo et al. 2022; Hashem et al. 2020; Nacka-Aleksic et al. 2022).

*Azadirachta indica* (neem), a Meliaceae tree widely recognized for its bioactive constituents, contains abundant ellagic, gallic, cinnamic, caffeic, ferulic, syringic, and salicylic acids, which collectively exhibit antioxidant, anti-inflammatory, hypoglycemic, immunomodulatory, and hepatoprotective properties (Hammadi et al. 2021; Liao et al. 2023; Yarmohammadi et al. 2021). While neem leaf extracts have improved growth performance, rumen fermentation, methane mitigation, and blood profiles in growing lambs and goats (Du Preez et al. 2023; Taethaisong et al. 2022), their application in reproductive stages, particularly late gestation, remains underexplored despite the demonstrated benefits of individual constituents like ellagic acid on antioxidant status and performance in sheep (Niu et al. 2024).

Given the capacity of polyphenols to enhance reproductive outcomes by alleviating oxidative stress and supporting metabolic resilience in ruminants (Bešlo et al. 2022; Hashem et al. 2020; Yang et al. 2022), supplementation with *A. indica* leaf extract during the periparturient period may mitigate gestational stressors. This work hypothesed to improve the ewes’ physiological health with the administration of lant extract during the gestation period. However, the present study, therefore, aimed to evaluate the effects of two doses of neem leaf extract, administered weekly during the final month of gestation, on hematological and serum biochemical parameters in ewes.

## Materials and methods

### Animals and treatments

Thirty pregnant crossbreed ewes of Texel, Suffolk, Hampshire, and Dorset, with an average age of 3.5 years and an average weight of 88 ± 3.2 kg. The production unit is semi-intensive, with all the ewes going out to graze at midday and returning in the afternoon. Their diet is based on sorghum and corn silage (70%) and commercial concentrate (30%, Purina, Cuautitlan, Mexico) formulated according to NRC (2007) nutrient requirements (Table 1), and within the facilities, they have access to chopped stubble bedding and water. Silage was prepared by the collection of whole maize plants (at medium stage with about 70% moisture content were chopped into 1–2 cm lengths using a forage chopper. Chopped maize was immediately filled into a flat with a 10-ton silo. After two months, experimental animals were initiated to be fed the silage.

Ewes were divided into three experimental groups (10 animals in one pen) and were given orally 0 (NE0), 20 (NE20), and 40 (NE40) mL of *Azadirachta indica* A. Juss leaf extract, every third day before the morning feeding during the last 30 days of the pregnancy period. The handling of animals was conducted in accordance with international bioethical standards and NOM-062-ZOO-1999.

**Table 1:** Chemical composition of the basal diet (commercial concentrate and corn silage)

Chemical composition	Concentrate	Corn silage
Dry matter^1^	880	360
Organic matter	325	684
Crude protein	157	106
Ether extract	120	85
Neutral detergent fiber	160	445
Acid detergent fiber	28	111
Lignin	8	18
Ingredient of concentrate, g/kg		
Corn grain flacked	200	
Corn grain cracked	260	
Sorghum grain	154	
Molasses	100	
Dried distillers’ grains with solubles	100	
Soya bean meal	96	
Wheat bran	70	
NaCOO_3_	10	
Mineral mix^2^	10	

^1^DM expressed as g/kg fresh silage.

^2^Mineral mixture: Ca, 190 g/d; P, 115 g/d; Mg, 63 g/d; Cl,167 g/d; K, 380 g/d; Na, 70 g/d; S, 53 g/d; Co, 3.3 mg/d; Cu, 197 mg/d;Fe, 360 mg/d; Mn, 900 mg/d; Se, 2 mg/d; Zn, 810 mg/d; Vit.A, 940(1000 IU/d); Vit.D, 165 (1000 IU/d); Vit.E, 374 (1000 IU/d).

### Preparation of extract

*A. indica* leaves were collected in the south of the state of Veracruz, randomly from several young and mature trees (minimum 5 different trees) in autumn, and were chopped (1–2 cm), and dried under the shade at 25–30 °C. The extract was obtained by infusing crushed *A. indica* leaves through soaking the chopped leaves in water in the laboratory at 25–30 °C for 48–72 h in closed flasks. After incubation, all flasks were incubated in a water bath at 39 °C for one hour and then immediately filtered, and the filtrate was collected and stored at 4 °C for further use (Salem et al. 2011).

### Sampling and measurements

Five milliliters of peripheral blood were collected from each ewe by jugular venipuncture in red vacutainer tubes (BD tube, Monterrey, Mexico) on days 1 and 30 of the experiment. The samples were conserved at room temperature until processing and were centrifuged at 1500 rpm for 10 min for serum extraction, which was stored in 1.5 ml Eppendorf tubes at-20 °C according to groups for further biochemical analysis.

Samples of both concentrate and silage were also collected during the experimental period and stored at -20 C for later chemical analysis. Two samples of the concentrate, silage, and *A. indica* extract were collected weekly during the 30 days of the experiment. Each sample type (i.e., concentrate, silage, or extract) was pooled and stored for further analysis.

### Sample analysis

The choice of biochemical measurements is conventionally used for diagnosing human and domestic animal hepatic and kidney damage, and general metabolic disorders. The serum samples were analyzedusing specific kits of IL ^TM^ tests for erythrocytes, hematocrit, hemoglobin, MCV (Mean Corpuscular Volume), MCH (Mean Corpuscular Hemoglobin), leukocytes, segmented neutrophils, lymphocytes, monocytes, basophils, eosinophils, plasma total proteins (#0018481300), creatinine (#0018480900), blood urea N (#0018480400), glucose (#0018480000), total blood protein, alanine aminotransferase or glutamic pyruvate transaminase (ALT/GPT; #0018480700), aspartate aminotransferase or glutamic oxalacetic transaminase (AST/GOT) (#0018480800), and alkaline phosphatase (ALP) (#0018480600).

All metabolites were determined using spectrophotometry analysis using a BTS 350 Chemistry System analyzer (Instrumentation Laboratory, Mexico).

Samples of concentrate and silage were analyzed for DM (#934.01), ash (#942.05), N (#954.01), and ether extract (EE; #920.39) according to AOAC (1997). The neutral detergent fiber (NDF; Van Soest et al. 1991), acid detergent fiber (ADF), and lignin (AOAC, 1997; #973.18) were analyzed using an ANKOM 200 Fibre Analyser Unit (ANKOM Technology Corporation, Macedon, NY, USA). The NDF was assayed without the use of α-amylase but with sodium sulphite in the NDF. Both NDF and ADF are expressed without residual ash.

### Identification of phenolic compounds by HPLC

The phenolic components of the aqueous extracts from *A. indica* A. Juss leaves were identified by HPLC (Agilent 1100, USA), which consists of a binary LC pump, a UV/Vis detector, and a C18 column (125 mm column length, 4.60 mm internal diameter, 5 μm particle size of the stationary phase inside the column, specifically the size of the silica particles coated with C18 (octadecyl) groups). The Agilent ChemStation was used to acquire and analyze the extracted chromatograms (El-Hefny et al. 2023; Lackner et al. 2025). A gradient mobile phase consisting of two solvents was employed: Solvent B [acetic acid in water (1:25)] and Solvent A (methanol). The gradient program was kept at a 100% B concentration for the first 3 min. After that, the eluent A concentration increased to 80% for the next 2 min, and then it dropped to 50% once more for the next 5 min at the 250 nm detection wavelength. Next, 5 min of 50% eluent A followed. Consequently, this mobile phase was used to confirm standard compounds and define the elution order of phenolic compounds (Eldesouky et al. 2024).

### Statistical analysis

Data were analyzed using the MIXED procedure of SAS (2002) with repeated measures (Littell et al. 1998). Terms in the model were diet (i.e., control (NE0, NE20, and NE40), and days of sampling (i.e., 1 and 30 of the experiment). Results are reported in tables and in text with their corresponding standard errors of the mean. Tests of simple effects were used to partition (slice) interaction effects by treatment in order to test the effects of period separately for each diet and the interaction treatment × time (SAS 2002). The significant differences between treatment means, time, and interaction treatment × time were determined with a value of significance of *P* < 0.05.

## Results

**Fig. 1:**
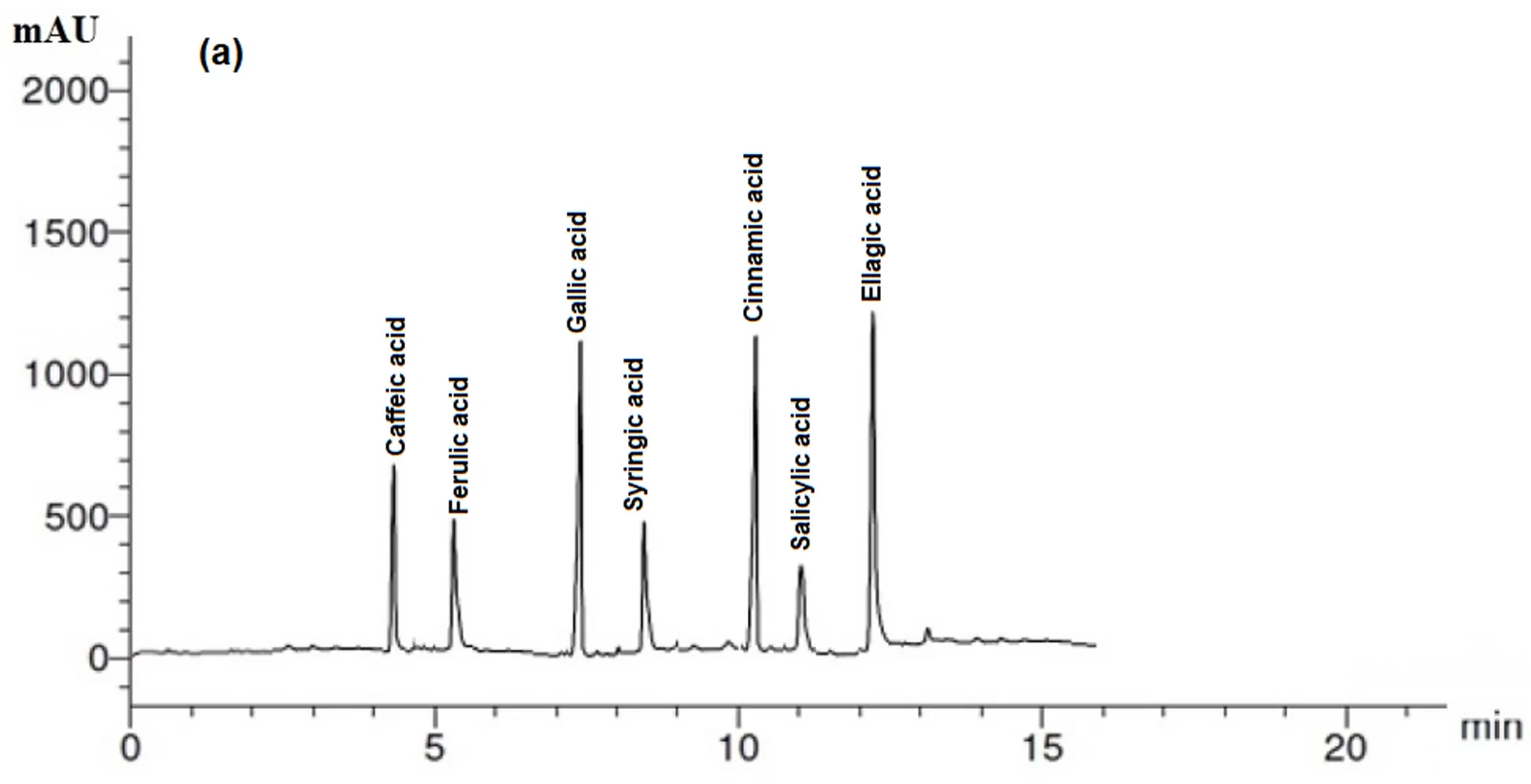
HPLC peaks of the identified phenolic compounds in the extracts from *Azadirachta indica* A. Juss

The results on High-performance liquid chromatography (HPLC) analysis of the *A. indica* leaf extract, as shown in Table 2; Fig. 1, revealed a diverse phenolic profile. The predominant compounds were ellagic acid, gallic acid, and cinnamic acid. Substantial concentrations were also detected for caffeic acid, ferulic acid, syringic acid, and salicylic acid, while the chlorogenic acid was not detected.

**Table 2:** Phenolic compounds of *Azadirachta indica* A. Juss

RT (min)	Compounds	Concentration (mg/mL)
4.20	Caffeic acid	8.66
5.10	Ferulic acid	6.24
7.00	Gallic acid	18.36
8.1	Chlorogenic acid	-
8.60	Syringic acid	5.04
10.00	Cinnamic acid	17.64
11.00	Salicylic acid	5.72
12.30	Ellagic acid	19.05

RT: Retention time (min).

### Hematological parameters

Hematological parameters in pregnant ewes during the last month of gestation, under weekly oral administration of 0 (NE0), 20 (NE20), or 40 (NE40) mL neem leaf extract, are detailed in Table 3. The results showed that erythrocyte counts exhibited significant main effects of treatment (*P* = 0.0187) and day (*P* = 0.0032), increasing from day 1 to day 30 in all groups, with higher values in NE20 and NE40 groups on day 30 compared to the NE0. Hematocrit and hemoglobin showed significant day effects (*P* = 0.0014 and *P* = 0.0054, respectively), declining over time across treatments, consistent with values remaining within or near normal ranges. Mean corpuscular volume (MCV) decreased markedly (treatment *P* = 0.0032; day *P* < 0.0001), with the most pronounced reductions in supplemented groups by day 30. Mean corpuscular hemoglobin displayed a significant day effect (*P* = 0.0006), increasing slightly over time. There was no significant interaction effect of neem leaf extract on any of these parameters.

Leukocyte parameters revealed targeted responses. Total leukocytes showed no significant effects. Among differentials, monocytes increased with treatment (*P* = 0.0261) and exhibited significant interaction (*P* = 0.0261), reaching 3.00 × 10⁹ cells/L in both supplemented groups on day 30. Eosinophils were elevated to NE40 (treatment *P* = 0.0453) but decreased over time (day *P* < 0.0001). Segmented neutrophils declined significantly by day 30 (*P* < 0.0001), while lymphocytes, basophils showed no major alterations.

**Table 3:** Haematological parameters^1^ in pregnant ewes, at the last month, and weekly orally administered with 0 (NE0), 20 (NE20), or 40 (NE4) Ml leaf extract of *Azadirachta indica* A. Juss, at days 1 and 30 of the experiment

Treatment	Normal range	Neem extract dose (mL/animal)^2^						SEM^3^	*P* value		
		NE0		NE20		NE40			Tret.	day	Tret. × Day
Day		Day 1	Day 30	Day 1	Day 30	Day 1	Day 30				
Erythrocytes, x 10^12^cells/L	8–16	8.08	8.63	8.22	10.05	9.16	10.12	0.341	0.0187	0.0032	0.3424
Hematocrit, %	27–45	44.20	39.40	48.50	42.80	46.90	42.70	1.686	0.0861	0.0014	0.9146
Hemoglobin, g/dL	80–160	144.20	131.10	154.60	142.30	155.50	142.00	5.126	0.079	0.0054	0.9938
MCV, femtoliters (fL)	23–48	61.40	45.20	57.80	42.20	50.90	42.00	1.825	0.0032	< 0.0001	0.1161
MCH, picograms/cell (pg/cell)	310–340	326.70	332.30	323.80	331.90	331.20	332.00	1.208	0.0786	0.0006	0.0837
Leukocytes, × 10⁹cells/L	4.0–12	10.17	9.60	10.99	9.11	9.74	11.90	0.941	0.5913	0.9032	0.1138
Segmentedneutrophils, × 10^3^cells/µL	0.7-6.0	37.00	3.31	36.80	2.95	36.60	4.78	1.771	0.9315	< 0.0001	0.8767
Lymphocytes, × 10⁹cells/L	2.0–9.0	5.63	5.45	6.11	5.07	7.11	5.76	0.594	0.294	0.1046	0.637
Monocytes, × 10⁹cells/L	0 to 0.75	1.80	3.00	2.30	3.00	2.80	3.00	0.110	0.0261	< 0.0001	0.0261
Basophils, × 10³ cells/µL	0 to 0.3	1.00	1.00	1.10	1.00	1.00	0.93	0.028	0.239	0.1694	0.5915
Eosinophils, × 10³ cells/µL	0–1.0	5.40	0.75	5.40	0.85	8.20	1.29	0.597	0.0453	< 0.0001	0.2117

^1^MCV, mean corpuscular volume (Volume Globulaire Moyen, VGM). which is a blood test measuring the average size of red blood cells; MCH, mean corpuscular hemoglobin, which is the average amount of hemoglobin in each red blood cell.

^2^Oral administration of *Azadirachta indica* A. Juss leaf extract at 0 (NE0), 20 (NE20), and 40 (NE40) mL.

^3^SEM, standard error of the mean.

### Serum biochemistry parameters

Serum biochemistry indicated metabolic shifts. Glucose demonstrated highly significant treatment (*P* = 0.0002) and day (*P* < 0.0001) effects, with a sharp decline by day 30 in all groups to hypoglycemic levels of 1.30, 2.10, and 1.70 mmol/L in NE0, NE20, and NE40, respectively; initial values were higher in supplemented ewes. Total blood protein increased in treated groups (treatment *P* = 0.0269; day *P* = 0.001). Plasma protein, urea, and creatinine remained stable without adverse changes. Alanine aminotransferase decreased over time (day *P* = 0.0006), while aspartate aminotransferase showed a day effect (*P* = 0.0118). Gamma-glutamyltransferase exhibited a significant interaction (*P* = 0.0101), with transient variations (Table 4).

**Table 4:** Serum biochemistry parameters^1^ in pregnant Ewes at the last month, and weekly orally administered with 0 (NE0), 20 (NE20), or 40 (NE4) Ml leaf extract of *Azadirachta indica* A. Juss, at days 1 and 30 of the experiment

Treatment	Normal range	Neem extract dose (mL/animal)^2^						SEM^3^	*P* value		
		NE0		NE20		NE40			Tret.	day	Tret. × Day
Day		Day 1	Day 30	Day 1	Day 30	Day 1	Day 30				
Plasma Protein, g/dl	60–80	79.60	80.00	75.60	77.00	79.50	79.00	1.792	0.1399	0.7769	0.8783
Creatinine, mmol/L	78–118	140.98	155.68	132.58	164.32	130.16	117.00	16.077	0.4539	0.5507	0.6083
Urea, mmol/L	4.0–7.0	3.99	4.15	4.12	4.39	4.21	4.51	0.222	0.4408	0.1961	0.949
Glucose, mmol/L	3.2–4.5	2.80	1.30	3.50	2.10	2.90	1.70	0.168	0.0002	< 0.0001	0.6781
Total blood protein, g/L	60–75	62.88	69.11	58.92	65.43	65.58	67.75	1.640	0.0269	0.001	0.3845
ALT, u/L	0–15	48.50	38.60	39.40	31.10	44.60	32.90	3.131	0.0524	0.0006	0.8785
AST, u/L	70–125	174.80	179.20	159.30	212.50	156.10	178.30	11.474	0.3335	0.0118	0.1517
GGT, u/L	10–40	71.00	40.50	61.70	70.90	71.10	66.60	6.008	0.1024	0.104	0.0101

^1^ALT, alanine aminotransferase or glutamic pyruvate transaminase; AST, aspartate aminotransferase or glutamic oxalacetic transaminase; ALP, alkaline phosphatase.

^2^Oral administration of *Azadirachta indica *A. Juss leaf extract at 0 (NE0), 20 (NE20), and 40 (NE40) mL.

^3^SEM, standard error of the mean.

## Discussion

### Phenolic profile of Neem leaf extract

The phenolic profile of the extract, featuring ellagic acid as the predominant compound, followed by gallic acid, cinnamic acid, caffeic acid, ferulic acid, salicylic acid, and syringic acid, with no detectable chlorogenic acid, is congruent with recent quantitative analyses of neem leaf extracts that highlight variability in chlorogenic acid content but consistent dominance of ellagic, gallic, and cinnamic acids (Alzohairy 2016; Hammadi et al. 2021; Yarmohammadi et al. 2021). These polyphenols exert synergistic bioactivities central to the observed physiological responses.

Ellagic acid, at the highest concentration, is recognized for its superior reactive oxygen species (ROS)-scavenging capacity, upregulation of Nrf2-mediated antioxidant enzymes Superoxide dismutase (SOD), catalase (CAT), and glutathione peroxidase (GPx), and inhibition of NF-κB-driven inflammation, thereby protecting erythrocyte membranes and supporting ovarian reserve function under oxidative stress, a mechanism highly relevant to the metabolic demands of late gestation (Bešlo et al. 2022; Niu et al. 2024; Yang et al. 2022). Gallic acid complements this by chelating transition metals, preventing Fenton reactions, and stabilizing red cell membranes, while also modulating apoptotic pathways in hepatic tissue (Hashem et al. 2020; Yarmohammadi et al. 2021). Cinnamic acid derivatives inhibit lipid peroxidation and exhibit antimicrobial properties that may indirectly support systemic redox balance in ruminants (Gessner et al. 2017; Hammadi et al. 2021). Caffeic and ferulic acids contribute to anti-inflammatory effects via cytokine suppression and α-glucosidase inhibition, respectively, underpinning glycemic regulation (Hashem et al. 2020; Taethaisong et al. 2022). Syringic acid neutralizes hydroxyl radicals and protects hepatocytes, whereas salicylic acid inhibits cyclooxygenase pathways, conferring aspirin-like anti-inflammatory benefits (Alzohairy 2016; Gessner et al. 2017).

### Hematological parameters in pregnant ewes, in the last month

The current investigation on three levels of weekly oral *Azadirachta indica* leaf extract supplementation during the final month of gestation in ewes and two sampling points (day 1 and day 30), revealed significant main effects of treatment and day on hematological parameters, with selective treatment × day interactions. The erythrocytes exhibited pronounced treatment and temporal effects, with no significant interactions for most indices. Erythrocyte counts (×10¹² cells/L) showed a significant treatment effect and day effect, with no interaction. On day 1, values were 8.08 (NE0), 8.22 (NE20), and 9.16 (NE40); by day 30, they rose to 8.63 (NE0), 10.05 (NE20), and 10.12 (NE40), indicating that supplemented ewes, particularly at 40mL, maintained higher counts throughout and exhibited greater increases over time. This contrasts with physiological hemodilution in late gestation, where erythrocyte counts typically decline (Chikhaoui et al. 2023; Yaqub et al. 2021; Yenilmez et al. 2021). The neem-induced elevation aligns with polyphenol-mediated protection against oxidative erythrocytic damage, as demonstrated in Kazakh sheep supplemented with ellagic acid (200–400 mg/kg), where RBC counts increased by 8–12% relative to controls through enhanced membrane integrity and reduced hemolysis (Niu et al. 2024).

Mean corpuscular volume (i.e., MCV) exhibited highly significant treatment and day effects, declining from day 1 values of 61.40 (NE0), 57.80 (NE20), and 50.90 (NE40) to 45.20, 42.20, and 42.00 fL on day 30, respectively, without interaction. This marked reduction, most evident in NE40 ewes, suggests a shift toward smaller, more oxygen-efficient erythrocytes, counteracting gestational macrocytosis observed in unsupplemented pregnant Yankasa and Kıvırcık ewes (Yaqub et al. 2021; Yenilmez et al. 2021). Similar MCV normalization has been reported with dietary polyphenols in heat-stressed ruminants, attributed to reduced oxidative swelling of red cells (Bešlo et al. 2022; Yang et al. 2022). Hematocrit and hemoglobin declined significantly over time across all groups, from initial means of 46–48% and 150 g/dL to 41–43% and 138 g/dL without treatment exacerbation, consistent with plasma volume expansion documented in late-pregnant Rembi and Akkaraman ewes (Balikci et al. 2007; Chikhaoui et al. 2023).

Leukocyte profiles indicated immunomodulatory influences. Monocyte counts revealed a significant treatment effect and treatment × day interaction, rising from baseline means of 1.80–2.80 to 3.00 × 10⁹ cells/L in both NE20 and NE40 groups by day 30, while remaining stable at 3.00 × 10⁹ cells/L in NE0 after an initial 1.80 × 10⁹ cells/L. This interaction reflects dose-dependent monocyte recruitment over time, consistent with caffeic and ellagic acid-induced phagocytic enhancement reported in goats fed anthocyanin-rich neem foliage (Taethaisong et al. 2022). Eosinophil counts were significantly affected by treatment and day, with NE40 ewes showing the highest values on day 1 (8.20 × 10³ cells/µL vs. 5.40 × 10³ cells/µL in NE0 and NE20) that converged toward 0.75–1.29 by day 30. This pattern may represent beneficial eosinophil priming rather than parasitism, paralleling polyphenol-driven modulation in Merino lambs supplemented with neem extracts (Du Preez et al. 2023). Segmented neutrophils declined markedly over time, potentially indicating reduced inflammatory tone under phenolic anti-inflammatory action (Gessner et al. 2017).

### Biochemical parameters

Glucose concentrations exhibited highly significant treatment and day effects without interaction. Values decreased from day 1 means of 2.80 mmol/L (NE0), 3.50 mmol/L (NE20), and 2.90 mmol/L (NE40) to severely hypoglycemic levels of 1.30, 2.10, and 1.70 by day 30, respectively. Supplemented ewes thus displayed higher initial glucose and a notably attenuated decline (–40% in NE20 vs. − 54% in NE0), mitigating the profound hypoglycemia characteristic of late gestation and pregnancy toxemia in ewes, where glucose often falls below 1.5 mmol/L in twin-bearing animals (Schlumbohm and Harmeyer 2008; Yenilmez et al. 2021). This stabilization is attributed to the inhibition of α-glucosidase by ferulic, gallic, and cinnamic acids, as well as the enhancement of GLUT4 translocation, as comprehensively evidenced in metabolic syndrome models and diabetic ruminants (Yarmohammadi et al. 2021; Hammadi et al. 2021).

Total blood protein increased significantly with treatment and over time, rising from day 1 means of 62.88 g/L (NE0), 58.92 g/L (NE20), and 65.58 g/L (NE40) to 69.11, 65.43, and 67.75 g/L, respectively, contrasting with typical gestational hypoproteinemia due to fetal uptake and hemodilution (Chikhaoui et al. 2023). This elevation in supplemented groups likely reflects reduced oxidative protein catabolism and enhanced hepatic synthesis under polyphenol-mediated Nrf2 activation (Hashem et al. 2020; Bešlo et al. 2022). Liver enzyme activities remained within physiological bounds, affirming safety. Alanine aminotransferase declined significantly over time, from initial means of 48.50 U/L (NE0), 39.40 U/L (NE20), and 44.60 U/L (NE40) to 38.60, 31.10, and 32.90 U/L, respectively, suggesting improved hepatic function rather than injury. Gamma-glutamyltransferase showed a significant interaction, with transient elevation in NE20 mid-experiment that resolved by day 30. These patterns corroborate hepatoprotective effects of ellagic and syringic acids via restoration of glutathione (GSH) and inhibition of Cytochrome P450 2E1(CYP2E1)-mediated oxidative damage, as observed in multiple livestock models (Alzohairy 2016; Du Preez et al. 2023; Yarmohammadi et al. 2021). Renal parameters (urea, creatinine) exhibited no adverse changes, further supporting the absence of toxicity at the administered doses.

## Conclusion

Weekly *Azadirachta indica* leaf extract supplementation at 20 mL during the last 30 days of gestation is recommended as the optimal regimen for pregnant ewes to elicit substantial beneficial modulations in hematological and biochemical profiles of late-gestational ewes through the concerted antioxidant, immunomodulatory, hypoglycemic, and hepatoprotective actions of its phenolic constituents. Thus, serving as a safe phytogenic intervention for ameliorating gestational oxidative and metabolic stressors in ruminants.

## Data Availability

Not applicable.
